# Refining electronic medical records representation in manifold subspace

**DOI:** 10.1186/s12859-022-04653-7

**Published:** 2022-04-01

**Authors:** Bolin Wang, Yuanyuan Sun, Yonghe Chu, Di Zhao, Zhihao Yang, Jian Wang

**Affiliations:** College of Computer Science and Technology, Dalian University of Technology, Dalian, China

**Keywords:** Electronic medical records, Distributed word representation, Geometric structure, Manifold

## Abstract

**Background:**

Electronic medical records (EMR) contain detailed information about patient health. Developing an effective representation model is of great significance for the downstream applications of EMR. However, processing data directly is difficult because EMR data has such characteristics as incompleteness, unstructure and redundancy. Therefore, preprocess of the original data is the key step of EMR data mining. The classic distributed word representations ignore the geometric feature of the word vectors for the representation of EMR data, which often underestimate the similarities between similar words and overestimate the similarities between distant words. This results in word similarity obtained from embedding models being inconsistent with human judgment and much valuable medical information being lost.

**Results:**

In this study, we propose a biomedical word embedding framework based on manifold subspace. Our proposed model first obtains the word vector representations of the EMR data, and then re-embeds the word vector in the manifold subspace. We develop an efficient optimization algorithm with neighborhood preserving embedding based on manifold optimization. To verify the algorithm presented in this study, we perform experiments on intrinsic evaluation and external classification tasks, and the experimental results demonstrate its advantages over other baseline methods.

**Conclusions:**

Manifold learning subspace embedding can enhance the representation of distributed word representations in electronic medical record texts. Reduce the difficulty for researchers to process unstructured electronic medical record text data, which has certain biomedical research value.

## Background

With the rapid development of medical information technology, hospitals have adopted a variety of medical information systems, including hospital information systems (HIS), clinical information systems (CIS), and radiology information systems (RIS). At the same time, EMR has also become popular. In recent years, a large number of clinical records have accumulated in medical institutions, and EMR data has increased rapidly. Huge opportunities have emerged from these data for health care audits, drug safety monitoring and clinical trials, etc.

When processing EMR data, we first need to represent words as real-valued vectors. For many biomedical natural language processing (BioNLP) tasks, such as Drug–Drug Interaction Extraction, Event Extraction, Protein-Protein Interaction Extraction [1–3], the word representation method is an important step. It turns out that effective word representations can help improve the performance of the BioNLP tasks. In recent years, distributed word representations have been widely used in the field of biomedical texts because they can better capture the semantic information of words. Distributed word representation uses the word co-occurrence to map the words into a low-dimensional dense vector, preserving the semantic information of the word. In this low-dimensional vector space, it is convenient to measure the similarity degree of two words according to the measurement methods, such as distance or angle between the vectors. Researchers apply distributed word representation to various NLP tasks.

Embedding words in a continuous semantic space has an important impact on many NLP tasks [4–6]. Mikolov et al. [7] used word co-occurrence to train word vectors iteratively and proposed the Word2Vec model. Jeffrey et al. proposed a Glove model considering local context features and global corpus features [8]. Wang et al. [9] trained word embeddings from clinical notes, literature, Wikipedia, and news, and used in biomedical NLP applications. Smalheiser et al. [10] proposed a word representation method based on word co-occurrence. Zhang et al. proposed a set of open biomedical word vectors/embeddings, BioWordVec [11]. Jiang et al. [12] proposed a new method for computing continuous vector representations that leverage deeper information to represent words. Jha et al. [13] leveraged the rich taxonomic knowledge in the biomedical domain to transformed input embeddings into a new space where they are both interpretable and retain their original expressive features. Chiu et al. [14] proposed a efficient method to align pretrained embeddings according to semantic verb clusters. Faruqui et al. [15] proposed a corpus-based approach that can be used to build semantic lexicons for specific categories.

The above word representation model has obtained good effects in the research of biomedical text and electronic medical record text. However, researches on the influence of the geometric structure of word vectors on the semantics of electronic medical records are insufficient. It is well known that the semantic information of words determines the representation of electronic medical record data. In cognitive psychology, these concepts are points in Euclidean space [16]. Words are mapped into low-dimensional dense vectors and exist in Euclidean space in the form of points. Therefore, in Euclidean space, the distance between words with similar semantics is smaller, while the distance between words with opposite semantics is larger. However, existing word representation models do not consider geometric information between words. As a result, human semantic similarity evaluation is not always consistent with Euclidean spatial metrics. Earlier psychometric studies have confirmed this conclusion. Tversky et al. studied whether the concept representation is consistent with the geometric sampling (GS) model and concluded that some hierarchical vocabularies are inconsistent with Euclidean embeddings [17]. The word vectors to be processed are regarded as points distributed in a high-dimensional semantic space, and the distance between the points is measured by Euclidean geometric straight-line distance. The linear structure of Euclidean space leads to cognitive biases in the word similarity, which requires a more efficient approach to deal with the similarity measure.

**Table 1:** Medical term pairs similarity on different methods

Medical term pairs	UMNRS-Sim(Ground truth)	Glove	Ours
P1: “peripheral edema”	sim(P1, P2) = 3.92	sim(P1, P2) = 0.55	sim(P1, P2) = 0.15
P2:“pulmonary edema”			
P3: “pkidney stone”	sim(P3, P4) = 4.69	sim(P3, P4) = 0.37	sim(P3, P4) = 0.32
P4:“ureteral obstruction”			

Table 1 shows the Similarity of two medical term pairs (“pulmonary edema”, “peripheral edema”) and (“ureteral obstruction”, “pkidney stone”) in the UMNRS-Sim, obtained through human judgment, Glove embedding with cosine similarity and our method. We can find that the results of ground truth and Glove are opposite. The reason is word vector generally exists in a high-dimensional semantic space by exhibiting a nonlinear structure. The word vectors to be analyzed and processed are regarded as points distributed in the high-dimensional Euclidean space [18], and the distance between the points is thus measured by the straight-line distance of the Euclidean geometry. This global linear structure of Euclidean space results in the cognitive bias for word similarity, which requires a more effective approach to handle space. The methods of Hasan et al. and Chu et al. solve the problem that the similarity of ground truth and Glove are opposite used the manifold learning [16, 19]. We also applied the manifold learning to obtain the similarity between the medical term pairs. It can be seen that the term pairs similarity results based on manifold learning is indeed consistent with the real similarity.

**Fig. 1:**
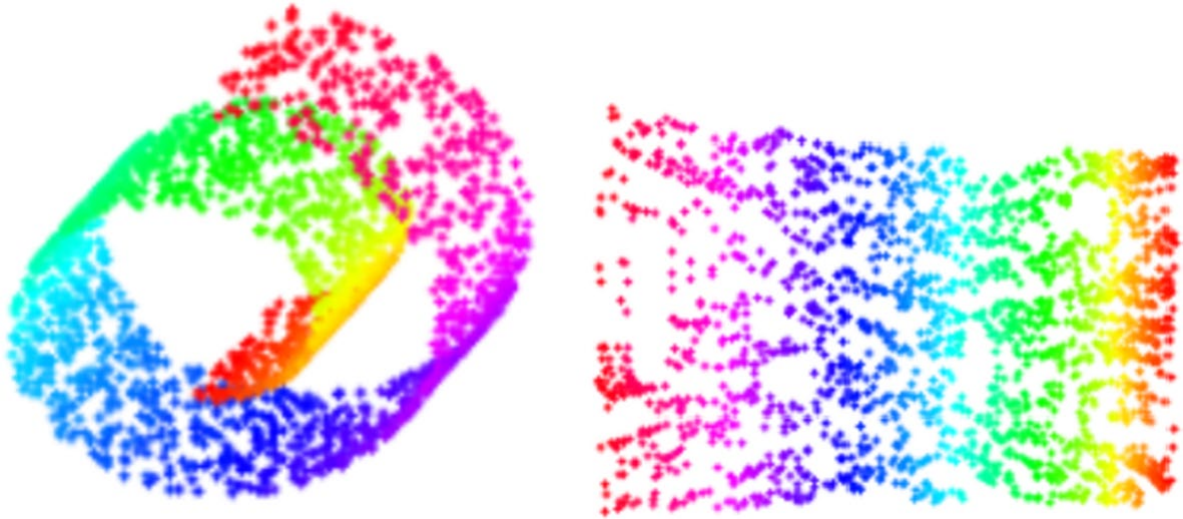
The relationship between high-dimensional space and low-dimensional embedding

Manifold learning tiles the sample distribution group in the high-dimensional feature space to a low-dimensional space. The sample distribution in the original space may be distorted. After tiling, it will be more conducive to the distance measurement between word vectors, and the distance will better reflect the similarity between the two samples. Figure 1 demonstrates that to map the original high-dimensional manifold space into the one in a relative low-dimensional embedding, which still preserves the structure in the original manifold space. Manifold learning estimates the distance between nearby terms by using direct similarity in the neighborhood, while the distance between faraway terms is approximated by multiple neighborhoods based on the shape of the manifold.

Manifold learning assumes that low-dimensional data is usually embedded in high-dimensional space [20–22], there be recovering the low-dimensional manifold structure of the data. There has been progress in the development of effective algorithms for processing nonlinear data and dimension reduction, such as isometric mapping Isomap [23], local linear embedding (LLE) [24] and its variations, and local tangent space alignment (LTSA) [25]. These algorithms include two common steps: learning the local geometry around each data point, and using the learned local information to non-linearly map the high-dimensional data points to the low-dimensional space.

In recent years, researchers have paid attention to the combination of pre-training word embedding and manifold learning. Manifold learning describes the local geometric structure information between sample points of word vectors by constructing adjacency graph structure of word vectors in high-dimensional space. Hashimoto et al. assumed that word representation and manifold learning were very suitable for recovering a Euclidean metric by the usage of co-occurrence counts and high-dimensional features. The manifold learning could be applied to embed words and phrases from high-dimensional space into low-dimensional space and its obtained word vectors should be regarded as the inputs of distributed word representation [26]. Hasan and Curry sampled an off-the-shelf word embedding to generate inputs as a manifold learning process that employed local word neighborhoods constituted in the original embedding space and re-embedded into a new embedding space by local linear embedding(LLE) of manifold learning [16]. By considering the effect of the matrix of the unfilled rank of each local neighborhood on the word representation, Chu et al. [19] imported MLLE to recover the word representation in a more general sense for improving the performance. In this work, we follow a methodology that adheres to this paradigm, Consider the nonlinear structure of EMR data, employs distributed word representation to train the biomedical word vector, which is used to learn a manifold to improve the results. This allows us to efficiently learn EMR data hidden semantic information, and we show that the model learns high-quality biomedical word representations. Specifically, we use the Word2Vec model to train word vectors on a specific corpus, then we use a manifold learning algorithm to re-represent the electronic medical record word vectors, and finally apply it to electronic medical record classification and text matching tasks. Solve the problem of irregularities in the structure and standardization of EMR data, which procrastinate the accuracy of medical text representation.

## Results

For intrinsic evaluation, we apply Pearson’s correlation coefficient and Spearman correlation coefficient to evaluate the effectiveness of different word embeddings. For different word embedding, we leverage cosine distance to measure the similarity of word pairs based on the learning word embedding. We explore several state-of-the-art methods to compare with our proposed method [11, 27–31]. Zhang et al. [11] proposed a BioWordVec method to train word embeddings by using biomedical text-domain knowledge. Chiu et al. [27] employed the Word2Vec model to train biomedical word embedding based on PubMed and PubMed Central articles. BERT has led to impressive gains on many natural language processing tasks [28]. A pre-trained biomedical language representation model for biomedical text mining (BioBERT) [29]. A lite BERT for self-supervised learning of language representations (ALBERT) [30]. An Empirical Study of Multi-Task Learning on BERT for Biomedical Text Mining (BlueBERT) [31]. The results in Table 2 show that manifold learning is valuable and useful in the task of improving word similarity in the biomedical domain. We note that the context pre-training model (such as BERT) lags other baselines on the word similarity task. BERT is optimized for specific downstream tasks that are not directly related to word similarity.

**Table 2:** Pearson and Spearman correlations coefficient score ($\times 100$) between model predictions and human ratings on three evaluation datasets

Method	MayoSRS		UMNSRS-sim		UMNSRS-rel	
	Pearson	Spearman	Pearson	Spearman	Pearson	Spearman
BERT	24.7	24.5	28.3	26.2	31.4	28.2
Zhang	62.5	61.1	64.9	62.5	57.0	57.0
Chiu	60.4	61.5	66.3	65.2	60.0	60.1
ALBERT	24.9	25.0	28.7	26.6	31.5	28.7
BioBERT	26.0	25.5	29.8	27.4	33.4	29.4
BlueBERT	26.5	27.6	31.2	28.9	33.9	30.4
Ours	**63.2**	**62.1**	**67.0**	**66.5**	**61.3**	**60.8**

Bold values denote the best result for each column of data

We use the Scikit-learn toolkit in the experiments [32]. We used Glove and Word2Vec to represent the word vectors, then we re-embedded word vectors using the MLLE algorithm. When using manifold learning to re-represent word vectors, we did not modify the word vector dimension but transformed between two equally-dimensional coordinate systems. When using MLLE to construct the neighborhood structure of the test words, we select a certain amount of words in the vocabulary obtained by Glove and Word2Vec as the training set. The training word window size is selected in the values of [1001, 1501, 2001] and the MLLE algorithm neighborhood value range is [300, 1000]. The results are listed in Tables 3 and 4.

In Table 3, we can find that our proposed method obtains the best results in the majority of evaluations of various indicators for medical coding classification. In addition to the relatively low performance of individual items, the performance of our method is outstanding with different parameters. Compared with convolutional neural networks (CNN) [33] and long short-term memory (LSTM) [5], the convolutional neural network and attention mechanism (CAML) [34] model produces the strongest results on all metrics under different categories of word embeddings. The success of CAML can be attributed to the attention of multi-label. For each label, the CAML uses a specific label weight matrix to generate attention for different labels of all the words in the text. We found that the performance of the method of adding different pre-training word vectors is better than that of randomly generating vectors, which shows the contribution of pre-training word vectors to medical coding classification. Compared with other pre-trained word vectors, our method yields certain advantages. This is because the geometric structures of word vectors, ignored by traditional distributed word vectors, imply the semantic information of the words. Noting that, we use manifold learning to represent the geometric structures between the words and integrated them into our model. Table 3 shows that compared with Word2Vec, our proposed method can generally improve the accuracy of different baseline models. We observed the BERT falls behind the other word embeddings on medical coding classification task. The possible reason is that the fine-tuning does not work well for high-dimensional structured prediction with a full label set that has more than 942 labels.

**Table 3:** Three basic models use different types of pre-trained word embeddings to predict performance

Method	Embedding	Macro AUC	Micro AUC	Macro F1	Micro F1	Test loss value	Top-10 recall
RNN	Random	0.854	0.972	0.204	0.653	**0.032**	0.772
	FastText	0.842	0.973	0.149	0.628	0.032	0.774
	Glove	**0.861**	0.974	**0.219**	0.656	0.031	0.788
	Word2Vec	0.851	0.974	0.165	0.642	0.031	0.783
	BERT	0.500	0.908	0.000	0.000	0.061	0.442
	ALBERT	0.503	0.915	0.026	0.018	0.054	0.446
	BioBERT	0.513	0.923	0.051	0.038	0.052	0.457
	BlueBERT	0.533	0.939	0.075	0.043	0.050	0.471
	Ours	0.857	**0.976**	0.182	**0.659**	0.030	**0.793**
CNN	Random	0.825	0.968	0.214	0.626	0.040	0.753
	FastText	0.665	0.921	0.012	0.223	0.053	0.488
	Glove	0.842	0.972	0.188	0.622	0.034	0.767
	Word2Vec	0.692	0.925	0.021	0.313	0.052	0.492
	BERT	0.549	0.906	0.000	0.000	**0.059**	0.442
	ALBERT	0.556	0.914	0.014	0.012	0.053	0.453
	BioBERT	0.559	0.921	0.015	0.041	0.047	0.459
	BlueBERT	0.567	0.929	0.021	0.047	0.042	0.464
	Ours	**0.852**	**0.974**	**0.217**	**0.628**	0.038	**0.779**
CAML	Random	0.855	0.978	0.257	0.656	0.032	0.806
	FastText	0.856	0.980	0.270	0.656	0.031	0.809
	Glove	0.867	0.978	0.272	0.647	**0.033**	0.801
	Word2Vec	0.855	0.980	**0.274**	0.662	0.030	0.813
	BERT	0.497	0.908	0.000	0.000	0.058	0.442
	ALBERT	0.505	0.916	0.026	0.022	0.054	0.457
	BioBERT	0.513	0.924	0.045	0.041	0.048	0.465
	BlueBERT	0.534	0.934	0.060	0.076	0.042	0.478
	Ours	**0.886**	**0.982**	0.270	**0.673**	0.029	**0.823**

Bold values denote the best result for each row of data(%)

Table 4 shows the results of our proposed method compared with the Glove model for the experiments on the clinical sentence pair similarity task. We used the Glove model by pre-training different corpora with correspondingly different dimensions. The dimensions of word embeddings in the experiments are 100, 200 and 300, respectively. We can see that our proposed method outperforms Glove. In the six billion word corpus, we obtained 69.4% of the Spearman rank correlation coefficient and Glove obtained 64.6% with 300 dimensions, which is an improvement of 4.8%. Meanwhile, in the six billion word corpus, our method got 67.0% and Glove got 64.6% with 300 dimensions, which is an improvement of 2.4% in this task. From Table 4, we can see that our proposed model outperforms baseline models in most cases, which also verifies the effectiveness of manifold learning in EMR data representation.

From the above results, we can see that all the performances of our proposed method are better than baselines. The main reason is our proposed model uses manifold learning to describe the geometric structure of EMR data word vectors. Manifold learning represents the local geometric structure information between sample points of word vectors by constructing the adjacency graph structure of word vectors in high-dimensional space. It will be more suitable to measure the distance between words and better reflect the similarity between samples based on the framework of the manifold.

**Table 4:** Average performance on clinical sentence pair similarity tasks

Space	Metric	Glove	Ours
6B300d	Pearson	69.2	**73.6**
6B300d	Spearman	64.6	**69.4**
6B200d	Pearson	69.9	**70.5**
6B200d	Spearman	64.6	**67.0**
6B100d	Pearson	68.3	**68.8**
6B100d	Spearman	**64.4**	63.5

Bold values represent the best result for each row of data. (window start $\in$ [0,1000], number of MLLE local neighbours = 500, manifold dimensionality = space dimensionality)

### Model interpretability

We evaluate the interpretability of our proposed approach. Table 5 is the top 10 words with the largest contribution for each corresponding medical code in the diagnostic summary. While the key-words study confirm by an expert. Classifier with CAML, using attention mechanism to calculate the weight of each word, the higher the weight, the greater the contribution of the word.

It can be seen from Table 5 that our method can obtain a higher keyword weight than Word2Vec. Through the word weight detection experiment in frequent diabetes medical codes, our method finds words that have important meanings in diabetes inference, such as “hemodialysis” “disease” and “diabetes”. While Word2Vec gives higher weight to the word “disease” rather than “hemodialysis” which is more directly related to diabetes.

From Table 6, experiments on the medical code of rare asbestosis medical through the manifold and the word with the highest weight in Word2Vec, we can see that our method finds several more relevant terms than Word2Vec, such as “pneumothorax” and “silhouette”. Compared with Word2Vec, our method can better find relevant terms and give a higher weight value, indicating that our method has higher interpretability.

**Table 5:** Words with the highest weight by manifold and Word2Vec for frequent diabetes medical code

Ours		Word2Vec	
Word	Weight	Word	Weight
Hemodialysis	0.7856	Disease	0.4320
Found	0.0235	Hemodialysis	0.2576
Disease	0.0347	Renal	0.0726
Stage	0.0043	Found	0.0123
Job	0.0052	Hypertension	0.0026
Hypertension	0.0071	Job	0.0010
Renal	0.0046	Stage	0.0009
Name	0.0083	End	0.0005
Mellitus	0.0008	Initial	0.0004
Diabetes	0.0005	Declared	0.0003

**Table 6:** Words with the highest weight by manifold and Word2Vec for rare asbestosis medical code

Ours		Word2Vec	
Word	Weight	Word	Weight
Pneumothorax	0.00535	Old	0.0617
Silhouette	0.0241	Service	0.0345
Mediastinal	0.0336	Evidence	0.0187
Opacity	0.0184	Partially	0.0171
Tissue	0.0173	Present	0.0162
Tobacco	0.0102	Without	0.0137
Meet	0.0085	Speaking	0.0095
Without	0.0091	Brief	0.0084
Remains	0.0075	Stable	0.0064
Partially	0.0059	Associated	0.0063

### Case study

**Fig. 2:**
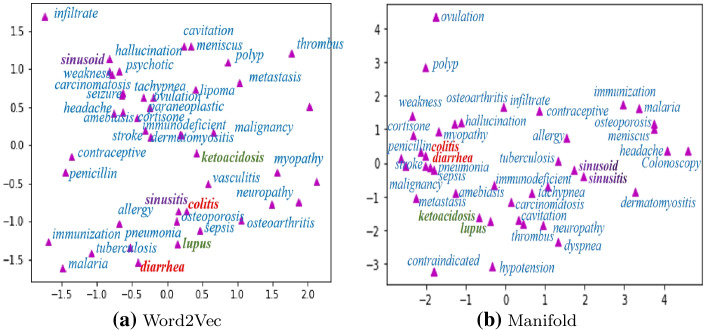
Visualization of word vectors on MayoSRS. The abscissa is the first dimension of vectors, and the ordinate is the second dimension of vectors

Figure 2 provides the similarity visualization of 43 words of biomedical domain in MayoSRS. The original 100-dimensional vectors are projected into a 2-dimenstional plane using TSNE toolkit.^1^ To visually show the performance of the manifold in our proposed model, we give some intuitive case studies comparing the word vectors processed by Word2Vec with the manifold learning post-processing, as is shown in Fig. 2.

We can see that through manifold representation, the medical term pairs with similar semantics are also close in Euclidean distance. For example in Fig. 2b “colitis” and “diarrhea” semantics are related, through manifold embedding, their Euclidean distance is also very close. However, in Fig. 2a Word2Vec embedding, the distance between the term pairs is faraway. Besides, the term pairs “sinusoid”, “sinusitis” and “lupus”, “ketoacidosis” with similar semantics are close in Euclidean distance after being represented by manifold. These cases show that manifold learning can capture the hidden semantic information of word vectors, which makes biological text representation more efficient and powerful.

## Discussion

Unstructured text data in EMR account for the vast majority, which results in EMR has such characteristics as incompleteness, unstructured, and redundancy. In the electronic medical record data representation, the existing distributed word representation model obtains the word vector through large-scale corpus training, ignoring the unstructured characteristics of EMR data and the influence of the geometric structure of the word vectors on the semantic information of the word. Therefore the electronic medical record data cannot be well represented. To address this problem, we introduce manifold learning into a distributed word representation model. We analyze the re-embedding word embeddings in terms of their principal components and demonstrated that the effectiveness of our proposed methods in the electronic medical record classification and text matching experiments. The experimental results show that the proposed model can effectively improve the performance of electronic medical record word representation and better capture its semantics.

### Effect of dimension

In our method, we start from a word embedding which is already a good embedding of the raw word co-occurrences. With the dimension of 300, our method exceeds the baseline method by Spearman coefficient with 1.6% and Pearson coefficient with 3.5%, respectively. Manifold learning usually starts from a high-dimensional original space and aims to reduce the number of dimensions. Therefore, the dimensions should be retained, otherwise, information may be lost during the calculation and selection of feature vectors in manifold learning. Table 7 show that under the condition that other parameters remain unchanged, the closer the of manifold learning dimension is to the original space dimension, the better the performance of re-embedding word vectors.

**Table 7:** The results of different dimensions on medical code classification between our method and Word2Vec

Dimension	Metric	Word2Vec	Ours
100	Pearson	**69.2**	68.8
100	Spearman	**63.8**	63.5
200	Pearson	69.2	**70.1**
200	Spearman	63.8	**64.3**
250	Pearson	69.2	**71.2**
250	Spearman	63.8	**65.6**
300	Pearson	69.2	**70.8**
300	Spearman	63.8	**67.3**

Bold values represent the best result for each row of data(%). (Original space dimension is 300d,(window start $\in$ [0,1000], number of MLLE local neighbors = 500, manifold dimensionality = space dimensionality)

### Effect of number of local neighbors

In the experiment, the number of neighborhood points directly affects the calculation speed, so selecting appropriate neighborhood points is an important issue for the algorithm. To study the influence of neighborhood on word embedding, we made quantitative analysis in the experiments. Table 8 gives the experimental results of different local neighbors on the medical code classification task. It can be seen that the optimal number of neighborhood points can be found for the experiments.

**Table 8:** The results of the different numbers of local neighbors on medical code classification between our method and Word2Vec

neighbor	Metric	Word2Vec	Ours
300	Pearson	**69.2**	68.5
300	Spearman	63.8	**64.2**
400	Pearson	69.2	**71.7**
400	Spearman	63.8	**65.6**
500	Pearson	69.2	**70.8**
500	Spearman	63.8	**67.3**
600	Pearson	69.2	**72.3**
600	Spearman	63.8	**68.2**

Bold values represent the best result for each row of data. (Space is Glove 840B 300d)

### Effect of window length

To investigate the effects of window length, we conduct the experiments based on the different window lengths. Without loss of generality, we use the Word2Vec model in the experiments. The results are shown in Table 9, we can find that we obtain better performance than Word2Vec on medical code classification tasks when the window length is higher. Through the experimental results of the window lengths, we can select the optimal starting position of the sliding window for each data set to re-embedding the word vector.

**Table 9:** The results of different window lengths on medical code classification between our method and Word2Vec

Win	Metric	Word2Vec	Ours
1000	Pearson	69.2	**70.8**
1000	Spearman	63.8	**67.3**
1500	Pearson	69.2	**71.9**
1500	Spearman	63.8	**67.1**
2000	Pearson	69.2	**71.2**
2000	Spearman	63.8	**67.3**
3000	Pearson	69.2	**70.7**
3000	Spearman	63.8	**66.9**

Bold values represent the best result for each row of data. (Space is Glove 840B 300d)

## Conclusions

In this study, we describe an unsupervised post-processing EMR data word re-embedding approach. EMR data is unstructured and has the characteristics of incompleteness. Defferent from the distributed word representation that ignores the influence of the geometric structure of the word vector, our proposed method imports the framework of manifold learning and renders off-the-shelf representations even stronger. To verify the effectiveness of the model mentioned in this article, we conduct experiments on electronic medical record data. Experimental results show that the algorithm proposed in this paper has achieved good results in both classification and text matching tasks, which is superior to other algorithms. Such a simple process could be applied as an initialization for pre-training the task-specific embeddings. In the future, we intend to extend our experiments to improve multilingual word vectors and other types of biomedical text data.

## Methods

**Fig. 3:**
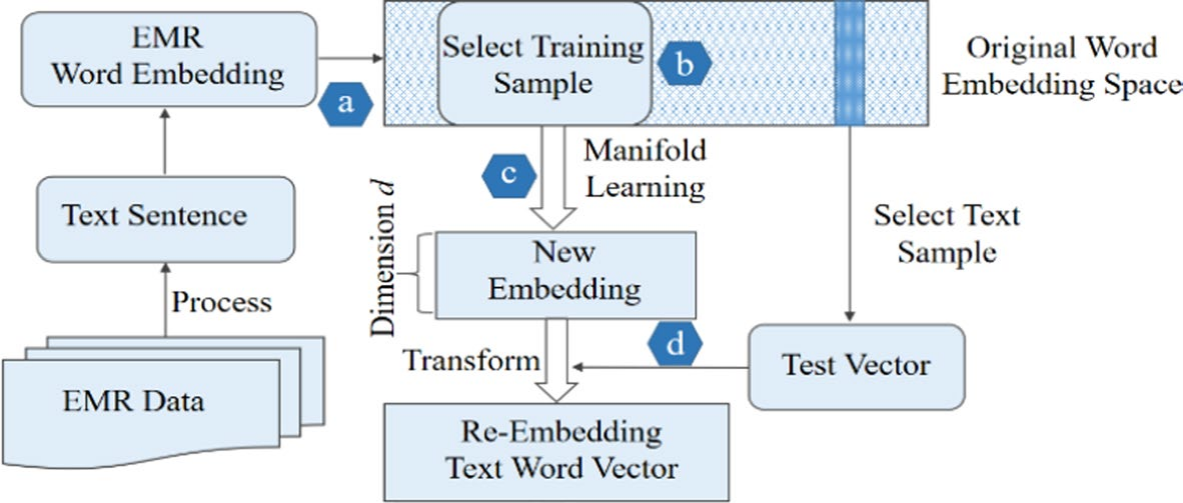
Biomedical word re-embedding via manifold learning

Our method aims to obtain a valid biomedical text representation based on word embeddings in the manifold framework. Manifold learning constructs the local structure of data vectors through adjacency graphs and restores the essential geometric structure of the data. The structure diagram of the model proposed in this paper is shown in Fig. 3.

The model in this paper can be divided into the following steps. In step (a), we obtain the processed EMR word representation vectors with the pre-training model. In step (b), we sample through a fixed window to train the manifold learning algorithm. In step (c), the manifold algorithm is employed to re-embed word vectors. In step (d), we fit the manifold learning algorithm to denote the word embedding in the specific task.

In step (a), specific field knowledge is included in the biomedical text, and the domain knowledge plays an important role in the representation of the biomedical text. To better represent the electronic medical record data, we use the Word2Vec and Glove models to train on the biomedical corpus to obtain pre-trained word vectors.

In step (b), we select a specific number of word vectors as the word vector window from the pre-trained word vectors in step (a). Hasan et al. deem that manifold learning attempts to restore a Euclidean metric [9]. Frequent words can better represent samples of the underlying space, thus restoring the manifold. While, all the word vectors are used to train the MLLE algorithm, which will generate a huge amount of computation. Therefore, we explore window sampling to train the MLLE algorithm. In the experiment, we conducted different window sizes on window sampling.

In step (c), we use the word vector window selected in step (b) to train the manifold learning algorithm MLLE. We extract the word vectors corresponding to the electronic medical record data from the pre-trained word vectors, and then we use manifold learning to map the word vectors contained in the electronic medical record data to the manifold space and re-embed the word vectors. Next, we introduce the training process.

For a given word vector set $X=\{x_1,x_2, \ldots , x_N \}$ , where *N* is the number of word vectors in the vocabulary, we use the *k* nearest neighbors to construct the neighbor structure of a word vector. The model constructs the word vector *X* and then represents the objective function as:

1 $$\begin{aligned} \begin{aligned} min\sum _{i=1}^N \sum _{l=1}^{s_i} {\parallel x_i-\sum _{j\in J_i}w_{j,i}^l x_j \parallel }^2 \end{aligned} \end{aligned}$$

Consider the neighbor set of $x_i$ with $k_i$ neighbors. Assume that the first $r_i$ singular values of $G_i$ are larger compared with the remaining $s_i=k_i-r_i$ singular values. Let $w_{i}^{(1)}, \ldots , w_{i}^{(s_i)}$ be $s_i\le k$ linearly independent weight vectors, which are defined as:

2 $$\begin{aligned} \begin{aligned} w_{i}^{(l)}=(1-\alpha _i)w_i(\gamma )+V_i H_i(:,l), l=1, \ldots , s_i \end{aligned} \end{aligned}$$

Here $w_i(\gamma )$ is the regularized solution, $V_i$ is the matrix of $G_i$ corresponding to the $s_i$ smallest right singular values, $\alpha _i=\frac{i}{\sqrt{s_i}}\parallel v_i\parallel$ with $v_i=V_{i}^{T}l_{ki}$, and *H* is a Householder matrix that satisfies $H_i=v_{i}l_{ki}= \alpha _i l_{s_i}$. We use the geodesic distance to calculate the neighbors of each word vector. The specific formula is as follows:

3 $$\begin{aligned} \begin{aligned} d_{ij}=\frac{f(x_i,x_j)}{\sqrt{d(x_i)\cdot d(x_j)} } \end{aligned} \end{aligned}$$

where $f(x_i, x_j)$ is the geodesic distance between $x_i$ and $x_j$, $d(x_i), d(x_j)$are the mean distances of $x_i$ and $x_j$ from other points, respectively. We use Lagrange to solve Eq. (1) to obtain the weight matrix *W*. Then, the weights are used to set up a new embedding *Y* of sample *X*:

4 $$\begin{aligned} \begin{aligned} E(Y)=\sum _{i=1}^N \sum _{l=1}^{s_i} {\parallel y_i-\sum _{j\in J_i}w_{j,i}^l y_j \parallel }^2 \end{aligned} \end{aligned}$$

In step (d), we re-embedded the word vector *x* obtained by the Glove model into the electronic medical record data using the model trained by Eq. (1). The formula is:

5 $$\begin{aligned} \begin{aligned} min\sum _{l=1}^{s_i} {\parallel x-\sum _{j\in J_i}w_{j}^l x_j \parallel }^2 \end{aligned} \end{aligned}$$

In Eq. (5), if $x_j$ is not in the K-neighborhood of the word vector *x*, then$w^{l}=0$. Transform *x* in *y* to which living in the new embedded space by the following equation:

6 $$\begin{aligned} \begin{aligned} E(Y)=\sum _{l=1}^{s_i} {\parallel y_i-\sum _{j\in J_i}w_{j}^l y_j \parallel }^2 \end{aligned} \end{aligned}$$

Eq. (6) is solved to obtain the optimal *y*, which is the re-embedding result of the word vector *x*.

The steps of the electronic medical record word embedding algorithm based on manifold learning are as follows:

**Table:** 

Algorithm: Electronic Medical Records Representation With Manifold Embedding.	
**Input**: Word set *X*, and threshold parameter *N*, *k*, and *d*.	
1.Using the Word2Vec and Glove models to train the electronic medical records obtain the word embeddings for each word.	
2.Select the word vector window from the pre-trained word vectors as the sample of manifold learning.	
3.The data samples obtained in step 2 are used to train the MLLE algorithm by using Eqs. (1) and	
(4) $X={X_1,X_2,\ldots , x_N}{\mathop {\longrightarrow }\limits ^{fit}}MLLE$.	
4.The MLLE model is trained using Eqs. (1) and (4), and then the model re-embeds the electronic	
medical records words embedding using Eqs. (5) and (6): $v(x)\rightarrow v^{'}(x)$.	
**Output:** Processed embeddings $v{'}(x)$.	

### Datasets

In this study, we carried out the experiments on four data sets. The UMNSRS and MayoSRS word similarity datasets are intrinsic metrics in the biomedical domain [35, 36]. We use a subset of UMNSRS-Sim and MayoSRS-Rel as our references, with 566 and 587 word pairs, respectively.^2^ The MayoSRS dataset is compiled from selected concepts from UMLS and includes 101 medical term pairs.^3^

MIMIC III is an open relational database, which contains all the records of the patient visits [37]. As the diagnostic information is merely considered in the previous research, we still only summarize the diagnostic information for each patient. A total of 52,722 diagnostic records were generated, and the average length of each diagnostic record was 1,596. In addition, we also converted uppercase words in diagnostic records to lowercase, removed punctuation marks, and characters with numbers. We listed all ICD-9 diagnostic codes for the diagnostic records according to the Bai’s method [38], and grouped them by the first three digits. A total of 942 medical codes were generated. on average, each visit has 11 medical codes. Given a discharge summary records, our goal is to predict associated medical codes. Therefore, medical code prediction is a multi-label text classification task. In multi-label text classification, we divide the data into the training set, test set, and valid set by a ratio of 7:2:1.

The dataset n2c2/OHNLP Track on Clinical Semantic Textual Similarity (ClinicalSTS)^4^ provides pairs of clinical text fragments, which are unrecognizable sentences extracted from clinical notes. The task is to assign a numerical score to each pair of sentences to express their semantic similarity. The scores are arranged in order, ranging from 0 to 5, where 0 means that the two fragments are completely different, and 5 means that the two fragments have complete semantic equivalence. There are 1,642 sentence pairs in the training sets, and 412 sentence pairs in the test sets.

### Evaluation metrics

To compare the performance of different algorithms, we use a series of evaluation criteria. For the multi-label classification problem, we used the following evaluation criteria, micro-averaged and macro-averaged F1 score and area under the ROC curve (AUC), the average loss value of the test set, and the average accuracy value and the top-10 recall score. The calculation formula of F1 as:

7 $$\begin{aligned} \begin{aligned} F1=\frac{2PR}{P+R} \end{aligned} \end{aligned}$$

where $P=\frac{True positives}{True positive+False positive}$ and $R=\frac{True positives}{True positive+False negatives}$ The calculation formula of AUC as:

8 $$\begin{aligned} \begin{aligned} AUC=\frac{\sum _{i\in Positive class}rank_i-\frac{M(1+M)}{2}}{M\times N} \end{aligned} \end{aligned}$$

where *M* is the number of positive samples, *N* is the number of negative samples.

The F1 value is an evaluation indicator, integrating precision and recall, used to reflect the overall indicator comprehensively. The micro-average is to summarize the category of all instances and calculate the average of all instance categories. Therefore, this metric is dominated in the medical code classification task. And the macro-average first calculate the value of each code separately, and then averages all the codes. Because the weight of frequent categories is the same as that of rare categories, the macro average metric is usually applied for rare medical code prediction. The top-10 roughly corresponds to the fraction of the top-n highest scored labels that are present in the ground truth. The metric is driven by potential use cases in computer-aided coding. It calculates the score of the top-n tags with the highest scores in the actual situation. The system recommends the top n codes for viewing by human experts.

For the evaluation criteria of word similarity, we used Pearson correlation coefficient and Spearman rank correlation respectively. Pearson correlation coefficient reveals the relationship between response characteristics and response. This method measures the relationship between variables Linear correlation. It is a non-parametric indicator that using the monotone equation to evaluate the correlation of them.

### Word embeddings

For the medical code classification task, we use Word2Vec to pre-train word vectors on the pending text of all discharge summaries, and then re-embed the obtained word vectors using manifold learning. Pre-trained embedding baseline methods include Random initialization(Random), Glove, Word2Vec,Fasttext, BERT, ALBERT, BioBERT and BlueBERT. For the word pairs similarity task, we use general publicly available Glove and Word2Vec embeddings as the original input. Word2Vec comes from Google’s pre-trained 300-dimensional news corpus. For out-of-vocabulary words, we randomly initialize according to the dimension size.

### Baseline classification model

In the medical code classification experiment, we employed three basic neural network models as baseline classifiers. The first one is a long short-term memory (LSTM) [5]. We first map the word in the diagnosis to a low-dimensional vector $emb\in R^d$ according to a pre-trained dictionary. Then, we input the word embedding sequence into the recurrent neural network:

$$\begin{aligned} l=LSTM(emb_1,emb_2,\ldots ,emb_n) \end{aligned}$$

The second one is the convolutional neural network(CNN) [33]. Like LSTM, we also convert the input sequence to word embeddings, and input them to the convolutional neural network:

$$\begin{aligned} l=CNN(emb_1,emb_2,\ldots ,emb_n) \end{aligned}$$

The third one is the combination of the convolutional neural network and attention mechanism (CAML) [34], which is currently the most advanced method in medical coding classification:

$$\begin{aligned} l=CAML(emb_1,emb_2,\ldots ,emb_n) \end{aligned}$$

For sentence pair matching, we use the ESIM model as a classifier. ESIM is a common basic model in sentence matching [39]. Like classification problems, we convert sentence pairs into corresponding sequence vectors:

$$\begin{aligned} Score=ESIM(sentence1, sentence2) \end{aligned}$$

The above models are treated as constants and the word vectors are variables. Our goal is to verify the effectiveness of the proposed method for improving biomedical text representations.

## Data Availability

The datasets generated and analyzed during the current study are available in http://rxinformatics.umn.edu/data/MayoSRS.csv and https://n2c2.dbmi.hms.harvard.edu.

## Abbreviations

EMR: Electronic medical records
HIS: Hospital information systems
CIS: Clinical information systems
NLP: Natural language processing
BioNLP: Biomedical natural language processing
GS: Geometric sampling
LLE: Local linear embedding
LTSA: Local tangent space alignment
MLLE: Modified locally linear embedding
LSTM: Long short-term memory
CAML: Convolutional neural network and attention mechanism.

## Footnotes

1 https://scikit-learn.org/stable/modules/generated/sklearn.manifold.TSNE.html.

2 http://rxinformatics.umn.edu/SemanticRelatednessResources.html.

3 http://rxinformatics.umn.edu/data/MayoSRS.csv.

4 The dataset is available at https://n2c2.dbmi.hms.harvard.edu.
